# Retrieval of carbon and inorganic phosphorus during hydrothermal carbonization: ANN and RSM modeling

**DOI:** 10.1016/j.heliyon.2024.e40999

**Published:** 2024-12-06

**Authors:** Abolfazl Shokri, Mohammad Amin Larki, Ahad Ghaemi

**Affiliations:** School of Chemical, Petroleum and Gas Engineering, Iran University of Science and Technology, Tehran, Iran

**Keywords:** Hydrothermal carbonization (HTC), Poultry litter (PL), Hydrochar (HC), Artificial neural networks (ANN), Optimization

## Abstract

In this study, modeling and optimization of Hydrothermal Carbonization (HTC) of Poultry litter were conducted to convert it into high-value materials. The aim was to understand the process and predict the effect of the influencing parameters on the product properties. The recovery of Inorganic Phosphorous (IP) and Carbon (C) was regarded as the model's response, although temperature and reaction time were thought to be important variables. Response Surface Methodology (RSM) was used along with temperature and time data sets ranging from 150 to 300C and 30–480 min, respectively, to identify the parameters influencing the response, three-dimensional networks, and optimization. Next, Multilayer Perceptron (MLP) and Radial Basis Function (RBF) were used to compare the results and improve the model fit. For these two neural networks, 7 neurons in two layers and 14 neurons in one layer were the ideal numbers. With fewer neurons and better accuracy and efficiency, the MLP model beat RBF with lower Mean Squared Error (MSE) values for both C (0.0015812 vs. 0.0037103) and IP (0.0014376 vs. 0.00623011) recovery and a higher R^2^ value (R^2^_C recovery_ = 0.99742, R^2^_IP recovery_ = 0.99816). These results demonstrate that MLP is a viable technique for maximizing resource recovery through HTC condition optimization, with potential uses in nutrient recycling and sustainable waste management. By examining the three-dimensional grids and obtained contours, it was found that temperature had a greater effect on the response, and the impact of time was more pronounced at lower temperatures. With increasing temperature and reaction time, C recovery decreased, while IP recovery increased. Furthermore, the optimal values for temperature and time were suggested to be 182.329 C and 427.746 min, respectively. The optimal product values under these conditions for C and IP recovery were obtained as 59.611 % and 29.114 mg/g, respectively.

## 1 Introduction

Livestock production is one of the fastest-growing sectors of agriculture, leading to an increased demand for food due to population growth. Intensive farming units, which are thought to be more efficient, have replaced grass-fed cattle in modern livestock production [1]. Yet, it needs to be disposed of appropriately because the buildup of litter presents a serious environmental risk [2]. Poultry farming produces the most Poultry Litter (PL) in concentrated locations, which makes up the biggest portion of all livestock waste globally [3]. Feces, urine, bedding, spilled feed, feathers, and other agricultural waste are collected to form PL, which is a heterogeneous substrate. Additionally, due to its higher nutrient and organic matter content than many other forms of biomass or waste, this material can be used primarily as a direct organic fertilizer in agricultural regions [4].

Although the direct application of PL in agricultural lands enhances soil fertility, environmental concerns emerge, such as the accumulation of soil elements and the leaching of pollutants into surface water [5]. In other words, the typical application of PL as a soil conditioner is deemed unacceptable because of its high nitrate content, which contravenes the European nitrate guidelines. This makes it crucial to provide a way to evaluate the worth of PL, like nutrition recovery, that is thought to be an effective way to deal with a number of problems within a unit. From an economic and environmental standpoint, the products that are produced as a result of PL conversion are quite valuable [6].

Hydrothermal carbonization (HTC), is a thermochemical process that uses water and regulated heat and pressure to treat biomass, including sewage sludge. The goal of this procedure is to create liquid and gaseous byproducts as well as hydrochar, a solid that is rich in carbon. Temperature, pressure, pH, water content, and residence time are important factors that affect HTC. Careful management is crucial since modifying these variables affects hydrochar's performance, energy content, and chemical characteristics. Sewage sludge HTC provides a sustainable waste management option [7]. HTC works especially effectively with high-moisture sludge since it reduces energy usage and does not require pre-drying. Because of its higher energy density, hydrochar made from sewage sludge shows promise as a fuel. The resultant hydrochar has several uses, such as adsorbent, soil conditioner, and renewable fuel [8]. HTC is a useful technique for cycle resource recovery since it also makes it easier to collect important nutrients like nitrogen and phosphorus, which are either discharged into the liquid phase or remain in the hydrochar. By deactivating microorganisms, such as SARS-CoV-2, found in sewage sludge, the HTC procedure also reduces health hazards, making it a safe treatment option in public health settings. Additionally, various biomass types like algae, lignocellulosic materials (like wood), and agricultural leftovers (like banana leaves) have been compared to sewage sludge [9].

Similar to sewage sludge, the calorific hydrochar produced by the HTC of algae can be used as biofuel. However, the type of biomass employed may have an impact on the energy yield. Hydrochar made from lignocellulosic biomass usually has less ash but still offers similar energy advantages. The recovery of nutrients, including algal nitrogen, which has potential for use in fertilizer, is further facilitated by the HTC of algae and agricultural leftovers. Phosphorus, a crucial ingredient for soil replenishment, can also be recovered from sewage sludge; however, its direct application in agriculture is limited due to its heavy metal content [10]. Various biomass kinds require different processing settings. For example, lignocellulosic materials may need higher temperatures for successful carbonization, while the HTC of algae functions best at moderate temperatures (180–250 °C). HTC is a promising thermochemical process that can produce useful products like hydrochar from a variety of biomass feedstocks, such as lignocellulosic biomass, algae, sewage sludge, and agricultural waste. HTC often improves energy density, makes nutrient recovery easier, and lowers the moisture content of biomass, while its parameters and effectiveness can fluctuate depending on the kind of biomass. HTC is especially beneficial for sewage sludge because of its high moisture content and substantial potential for nutrient recovery [11]. This method saves energy because it doesn't require pre-drying, especially for raw materials like sludge that have a high moisture content. It provides health benefits by successfully inactivating viruses, including SARS-CoV-2. Nitrogen and phosphorus, which are useful for agriculture, are retained by HTC; nevertheless, the presence of heavy metals in the sludge frequently limits their use. Achieving uniform heating requires maintaining controlled humidity, particularly in materials with different water contents [12].

Certain temperature conditions are necessary for the best processing of certain raw materials. While lignocellulosic biomass may require greater temperatures, algae and sewage sludge require moderate heat. Dehydration and decarboxylation activities cause hydrochar's carbon concentration to rise. As long as the metal content is appropriately controlled, hydrochar can be used as a sustainable fuel, absorbent, and soil conditioner, especially in agriculture. Products from HTC reduce waste and recover nutrients, making recycling easier. HTC also lowers microbial hazards, which makes it a useful tool for managing wastewater, particularly during epidemics. Different biomass species, including algae, sludge, and agricultural waste, produce hydrochar with unique qualities that enable customization for certain uses [13].

Wet biomass waste, such as food waste and municipal solid waste (MSW), can be efficiently converted into energy-dense products like charcoal via HTC, which is used in waste management and renewable energy. From the standpoint of energy recovery, wet biomass is efficiently converted into coal—a high-carbon, energy-dense product similar to lignite—by HTC. Compared to more conventional techniques like incineration, HTC is a more energy-efficient choice for handling high-moisture waste because it eliminates the need for pre-drying [[14], [15], [16], [17]].

By storing carbon in solid form and reducing methane emissions from landfills, HTC lowers greenhouse gas emissions. Additionally, this process produces nutrient-rich byproducts that can be applied to agriculture, improving soil health and fostering a circular economy [18]. The economic viability of HTC, especially its potential to generate biochar that can rival conventional fossil fuels, is one of its benefits in advancing economic sustainability. This makes HTC a viable economic option to improve energy self-sufficiency and lessen reliance on imported fuels, particularly when scaled. HTC end products, including as liquid and gas by-products, can help produce biogas and other sustainable energy sources.

Additionally, in industries where coal is frequently used, like energy, cement, and manufacturing, biochar made from HTC can be used as a fossil fuel alternative. HTC's by-products, such hydrochar, which may be applied as soil supplements, support sustainable agriculture [19]. A sustainable agricultural system is supported and plant development is greatly increased when hydrochar and compost are combined, according to research. HTC has a holistic approach to waste management and renewable energy, tackling sustainability, economic, and environmental objectives all at once. This technology is a promising instrument for attaining a sustainable energy future because it not only provides a sustainable waste treatment solution but also produces feasible energy sources [[20], [21], [22]].

Relying on the quantities, characteristics, and uses of the intended goods, various methods can be used to create goods with better qualities than pure PL. One example is thermochemical reactions. The thermochemical processes of gas production, pyrolysis, combustion, and HTC are frequently employed [23]. Every technique has disadvantages. The presence of PL with a high percentage of moisture has a detrimental effect on the process because the majority of operational processes employ dry raw materials [24]. Therefore, the HTC process, which is a thermochemical conversion process and does not require drying of raw materials (resulting in lower energy consumption), can be utilized. HTC is a promising option compared to other methods because it can convert wet materials into valuable biofuel products, offering advantages such as reduced volume and mass [25].

The HTC process can be used as one of the effective PL heat treatments to produce valuable products with multiple applications in agriculture and the environment, such as recovering Inorganic Phosphorous (IP) and mitigating environmental issues [26]. Thus, HTC is referred to as a pressured thermochemical procedure that uses pressure and medium temperatures (180–260 (c)) to transform biomass and/or biowaste with a high moisture content. The feedstock macromolecules go through hydrolysis, dehydration, decarboxylation, aromatization, and recondensation processes as part of the many chemical events that make up the HTC method. These reactions lead to the production of a solid product (Hydrochar (HC)), a liquid byproduct, and a gas, each with distinct distribution, composition, and structure. The outcomes depend on the treatment conditions [27]. Finding and improving the critical factors influencing the HTC procedure is essential to producing more useful and valuable end products. Process conditions like temperature and reaction time are examples of these crucial factors. A valuable carbon material obtained through the HTC process that has garnered significant attention is the HC product. Some of the studies conducted are summarized in Table 1.

**Table 1:** The literature review of the application of models for predicting C recovery (%) and IP recovery (mg/g).

Researcher	Description	Results	Ref.
Kumar et al.	Modeling of the Batch Sucrose crystallization kinetics using ANN	In comparison with conventional regression analysis, the ANN model provides more accuracy	[28]
Kumar et al.	Neural network prediction of interfacial tension at crystal/solution interface	An ANN is used to create the model first, and the kriging interpolation technique is subsequently used to improve it	[29]
Ghanim et al.	They examined the impact of process parameters using a hybrid ANN model	They discovered that the ANN algorithm could accurately forecast the HTC therapy procedure	[30]
Ismail et al.	They utilized an ANN-Kriging hybrid model to predict C and IP recovery in HTC.	Their findings indicated that the new ANN-Kriging hybrid model provided higher accuracy in comparison to a hybrid model	[31]

Because of its nutrients and resistant biological carbon, HC can supply essential nutrients to plants that are necessary to increase soil fertility [32]. However, treatments can have a significant impact on a hydrocolloid's nutritional content. Phosphorus (P) is one of the most important minerals for the growth of both plants and animals. Even with a great deal of experimental study on turning PL into products with significant value, the technique is still difficult to explain [33]. It is thought to be difficult to determine how process factors relate to product attributes. Process modeling is required for industrial-scale forecast, design, and optimization in order to make the HTC process easier to grasp [34].

One promising technique for the long-term recovery of C and IP from biomass is HTC. Despite the fact that earlier research has looked into a variety of modeling approaches to forecast HTC results, comparative analysis of the prediction accuracy of various models—particularly with regard to C and IP recovery—is lacking. Three significant contributions to HTC research are made by this study: The Multilayer Perceptron (MLP) model is the most accurate and efficient for predicting C and IP recovery, with a significant reduction in Mean Squared Error (MSE) values, according to a thorough comparison of the MLP, Radial Basis Function (RBF), and Response Surface Methodology (RSM) models. This study gives important insights into the mechanisms underpinning HTC and offers recommendations for optimizing conditions based on desired recovery results by examining the effects of temperature and reaction time on C and IP recovery. With possible uses in carbon sequestration, nutrient recycling, and sustainable waste management, the results show that enhanced HTC methods can be a practical approach to resource recovery. A foundation for future research and industrial use of HTC as a waste-to-resource technology is laid by these contributions, which together improve the predictive modeling of HTC processes. The primary objective of this work is to use the HTC process to create a reliable model that can forecast and characterize the changes in IP and C recovery in HC by PL. Using RSM software and interactive three-dimensional networks of response input parameters, a suggested model was created in the first step. In the next step, these results were optimized using ANN, including MLP and RBF, and the modeling results were compared with each other. This model examines the effect of two input parameters, namely temperature and reaction time, on responses that include IP and C recovery. Finally, the model was optimized, and the optimal values of the input parameters were reported.

## 2 Materials and methods

For this study, PL samples from a farm in Ireland were used for carbonization experiments. One kilogram of weight every sample was kept in polyethylene bags at −80 °C in a refrigerator for reuse afterward. The HTC pretreatment samples that were saved were used exactly as they were received. The PL was completely broken filtered into particles smaller than 0.75 (mm), and dried in an oven set at 105 (c) for 24 (h) before to decomposition. An 8.0 L Parr stirred SS316 pressure reactor with a detachable glass liner was used for the studies. An electric heater supplied the heat required to maintain the reactor's temperature. The purpose of the first HTC trials was to examine the effects of residence duration and treatment temperature. With a moisture content of 37.25 ± 2.53 %, the glass liner was usually loaded with PL (ar) (200.51 ± 0.30 g). After adding 1 L of distilled water, the mixture was thoroughly stirred for half an hour. The glass liner holding the suspension was put into the stainless steel reactor after the mixture's pH was measured at 8.83 ± 0.042. To remove any remaining air, the reactor was shut and continuously purged with nitrogen for 10 min. Following the removal of the air, the reactor was heated to the target treatment temperature (150 ≤ T ≤ 300 °C) and kept there for a predetermined amount of time (30 ≤ t ≤ 480 min). After stopping the heating, the reactor had been immersed in an icy bath of water to rapidly cool it to the ambient temperature. Following filtering by vacuum to recover the hydrochars as a solid residue, they underwent multiple cleanings with distilled water and acetone before being dried at 105 °C for an entire night. After being homogenized and crushed to a size of less than 0.75 mm, the hydrochars were kept for examination at room temperature in an airtight container. The soluble chemical components and liquid by-product were kept at 4 °C in a closed container. The hydrochar and liquid by-product samples' identifications (IDs) are expressed as t-T, where t stands for time and T for temperature.

### 2.1 Data collection

With (t) standing for time and (T) for reaction temperature, the resultant HC samples are denoted as (t-T). For every experiment, IP and C were generated following the recovery and measurement of HC. A portion of the experimental data gathered for each trial is displayed in Table 2.

**Table 2:** Input and output data used in this study.

A: Time (min)	B: Temperature (c)	R_1_: IP (mg/g)	R_2_: C recovery (%)
30	150	7.76	90.92
30	175	9.72	76.29
30	200	17.12	50.88
30	225	35.46	46.31
30	250	45.17	42.83
30	275	59	31.37
30	300	49.06	40.23
120	150	16.33	81.8
120	200	24.36	56.41
120	225	41.59	43.54
120	250	43.51	44.14
120	275	45.29	39.36
120	300	53.4	32.71
480	150	16.53	76.24
480	200	33.07	49.44
480	225	46.2	41.58
480	250	47.84	43.77
480	275	47.83	36.85
480	300	57.76	27.3

### 2.2 Model design

**Fig. 1:**
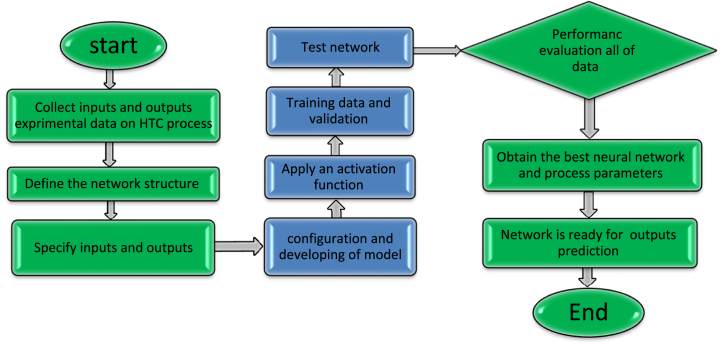
The ANN model's design for HTC's C and IP recovery predicted.

At first, after determining the input and output parameters of this study (as shown in Table 2), modeling is conducted [35]. Backpropagation learning methods depending on MLP and RBF were used to model this data. Three-dimensional networks that show how interaction factors affect their reaction have finally been recovered [36]. As demonstrated in Eq. (1), neural net inputs are specified prior to network construction [37]:

(1) Response=f(Time,Temperature)

in the next step, the normalization operation was performed due to the differences in weights and slopes. Otherwise, learning in neural networks will progress slowly [38]. To avoid this issue, Eq. (2) can be used for normalization [39]:

(2) $$X_{i\_\text{normal}}=2\left(\frac{x_{i}‐x_{\min}}{x_{\max}‐x_{\min}}\right)‐1$$

where X_max_ and X_min_ indicate the maximum and minimum values, and X_i_ stands for any input or outcome value [40]. As a result, every input and result range falls within −1 and +1. Once the network learning technique has been specified, training information is used to figure out network scales and variables and enhance efficiency [41]. In the network learning analyze, a set of training data is used to improve the model using verification information, and then test data is used to evaluate the model. The accuracy and performance of the ANN-based model were assessed using statistical indicators including Mean Squared Error (MSE) and correlation coefficient (R^2^) in Eqs. (3), (4)). These criteria are used to evaluate how accurate the projected data are in comparison to the real numbers [42].

(3) $$\text{MSE}=\frac{1}{n}\sum_{i=1}^{n}{\left(Y_{\text{Pred}}‐Y_{\text{Real}}\right)}^{2}$$

(4) $$R^{2}=\sum_{i=1}^{n}\left(\frac{{\left(Y_{\text{Pred}}‐Y_{\text{Real}}\right)}^{2}}{{\left(Y_{\text{Pred}}‐Y_{\text{Mean}}\right)}^{2}}\right)\;$$

in this model, a set of input-output pairs is used for training. In this setup, the input variables are treated as independent variables, while the output variables are treated as dependent variables [43]. During training, learning this model involves repeatedly adjusting its parameters and determining the relationship between input and output variables [44]. The goal of this model is to minimize the difference between predicted and actual output to ensure accurate predictions on unseen data. The goal is to generalize this model to new, unseen data by learning from existing data. Therefore, when the model's performance is satisfactory, it can be used to predict new inputs [45]. In this study, MLP and RBF models are used to predict the values of indicators related to HTC. In general, the methodology of this study is illustrated in Fig. 1.

### 2.3 Artificial neural network (ANN)

A common framework for transmitting information among both input and outcome data is the artificial neural network (ANN). An ANN is made up of many coupled inputs and outputs via neurons in layers that are hidden [46]. One form of machine learning that imitates the kind of neurons found in the human brain is neural networks. Either linear and non-linear transfer functions can be employed for instruction and learning in this kind of neural network [47]. The outcome layer usually uses linear functions, but the deep layers utilize non-linear functions. Since neural network learning accounts for 80 % of the input data, this section is essential [48]. The inputs from the neurons of the layers that are concealed are passed via internal processes to produce the desired output. Neurons in neural networks connect to one another via signals. MLP is a particular kind of ANN that uses backpropagation procedures to learn complex data [49]. Creating ideal weights across neurons serves as one of the neural network's objectives. Actually, each neuron contributes its received data to the threshold or bias value after multiplying it by the weight [50]. Lastly, the intended output is produced if the outcome goes above the predetermined boundary; if not, the determined weights are corrected. With W_i_, b, and X_i_ standing for weight, bias, and input data to the neuron, respectively, Eq. (5) provides a summary of the input data process [51]:

(5) $$net=\left(\sum_{i=1}^{n}w_{i}x_{i}\right)+b$$

Calculating a neuron's response in a neural network is demonstrated by Eq. (6):

(6) y=f(neworkt)

#### 2.3.1 Multilayer perceptron (MLP)

An MLP has a minimum of three layers—an input layer, one or more hidden layers, and an output layer—with a sizable number of neurons. The signals sent across the connections between neurons and their associated weights have a major impact on each one of them [52]. In order to support the process of education throughout the network, learning algorithms are utilized to process the inputs and datasets [53]. Each neuron multiplies the weights in its provided data and records the outcome, per Equation (7). Subsequently, this value has been added to the bias and displayed as the final value in accordance with Eq. (8) [51]:

(7) $$\phi_{K}=\sum_{i=1}^{N}w_{\text{ki}}x_{i}$$

(8) $$\text{out}=f\left(\phi_{k}+b_{k}\right)$$

**Fig. 2:**
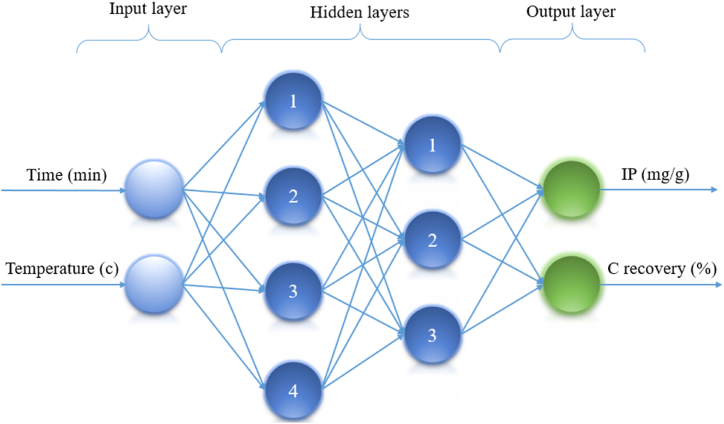
MLP's schematic layout in this investigation.

The mechanism of a neuron involves a linear combination of input signals, a bias, an activation function, and an output signal. In the equations, x_i_ represents the input signals, w_ki_ represents the connection weight, b_k_ is the bias term, and ϕ_K_ represents the activation function. Neurons are in charge of producing signals of output [49]. An MLP neural network in the present research has four layers: an input layer, two layers that are concealed, and an output layer, as demonstrated in Fig. 2 [54]. The initial layer employed two neurons as input, followed by the primary hidden layer with four neurons, the following hidden layer with three neurons, and the last layer (response) with two neurons. For the output layer in this network, the Pureline activation function is taken into account [55].

#### 2.3.2 Radial basis functions (RBF)

**Fig. 3:**
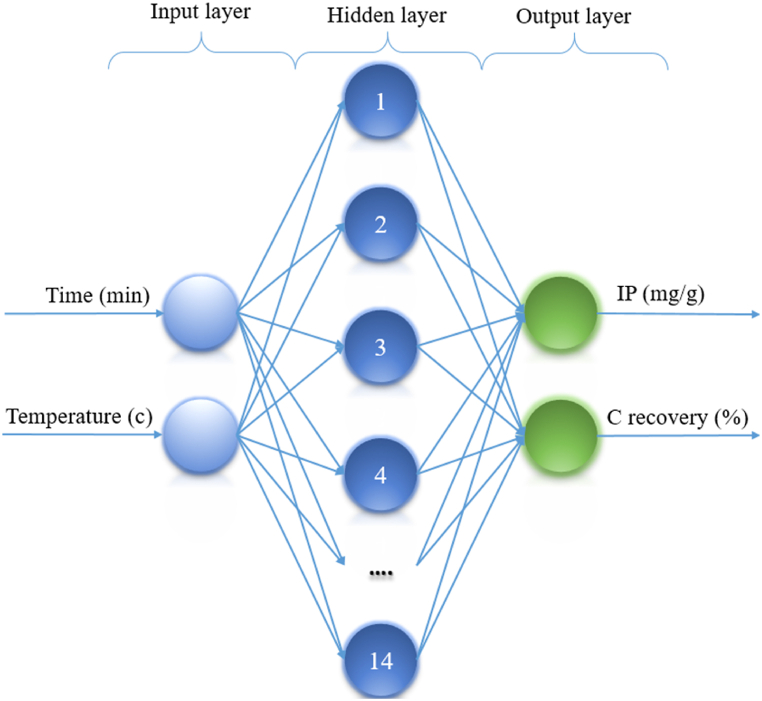
The RBF's schematic structure in this investigation.

The benefits of RBF, which was first presented around 1988, are its simplicity and universality. They are more attractive than feedforward networks because of their simplicity, which eliminates time-consuming and pointless computations [56]. Girossi, Poggi, Hartmann, and Kepler subsequently claimed that RBF networks are highly efficient approximations at this point. Any continuous function can be successfully predicted by this network if the hidden layer contains a sufficient number of neurons. A few more benefits of the RBF network are that its characteristics may be easily estimated and physically interpreted [57]. As shown in Fig. 3, RBF consists of three layers: input, hidden, and output. Each neuron in the hidden layer has a distinctive center and radius for the radially operation, and the number of neurons can be as high as the number of data points. A linear mixture of the hidden layers' properties is computed in the end result [58]. In contrast to MLP feedforward networks, this network may employ a greater amount of neurons in the layer that is hidden.

Training the centers and widths of the radial functions and learning the weights between the neurons of the hidden and output layers are the two phases of learning in the RBF neural network. The trial-and-error method is used to choose the number of neurons in this network [59]. While concentrating on the ideal error model, the process first starts with an enormous amount of neurons and then progressively decreases their number. Eq. (9) presents an overall form of the desired estimate function in RBF neural networks [60]:

(9) $$f\left(x\right)=\sum_{i=1}^{m}w_{i}\phi_{i}\left(x\right)$$

where ϕ_i_(x) is an indicator of the distance from the point of origin, as given by Eq. (10), and W_i_ is the weight across neurons:

(10) $$\phi_{i}\left(x\right)=\phi \left(\left\|x‐c_{i}\right\|\right)$$

The center of functions is denoted as Ci. The RBF neural network uses the least squares criteria as its goal function and the Gaussian function described in Eq. (11) as the network's activated function [49]:

(11) $$\phi \left(x\right)=ae^{‐\frac{{\left(x‐b\right)}^{2}}{2\sigma^{2}}}\;\sigma >0$$

The values of the coefficients “a", “b", and "σ" are used here. While “b" indicates the peak's exact center and "σ" (standard deviation) indicates the bell's amount of stretch or growth, the constant “a" determines the curve's maximum height [57].

### 2.4 Response surface methodology (RSM)

Table 3 displays the lowest and highest values for the two groups of data that are identified as the inputs and outcomes in the present investigation.

**Table 3:** RSM's upper and lower limit data values.

	Time (min)	Temperature (c)	IP (mg/g)	C recover (%)
Min rowhead	30	150	7.76	27.3
Max rowhead	480	300	59	90.92

In RSM software, one can effectively predict the response using a quadratic model. This software's suggested model relies on Eq. (12), in which Y stands for the response and X_i_, X_j_, …, X_k_ are the input parameters that affect it [61]. ε stands for the accidental error, β_0_ for the point of intercept term, β_i_ for the linear impact, β_ii_ for the squared force, and β_ij_ for the reciprocal effect (where j = 1, 2, etc.) [58].

(12) $$Y=\beta_{0}+\sum_{i=1}^{n}\beta_{i}x_{i}+\sum_{i=1}^{n}\sum_{j=1}^{n}\beta_{\text{ij}}X_{i}X_{j}+\sum_{i=1}^{n}\beta_{\text{ii}}x_{i}^{2}+\varepsilon$$

Three-dimensional layouts are used to assess the RSM findings. The criteria listed in Table 2 are used to establish the signs, answers, and data ranges.

## 3 Results and discussion

### 3.1 Analysis of variance (ANOVA)

Initially, experimental design software version 13 was utilized to model and identify the parameters influencing the response [58]. The data was inputted into the software in the format of Table 2. Following the modeling process, 2 s-order Eq. (13) and Eq. (14), were generated for the two outputs [62]. Valuable information, such as Analysis of variance (ANOVA) tables, determination of parameters affecting the response, 3D networks, and optimization, can be obtained using this software. The first step is ANOVA analysis. P-values in this table show how important each of the response's variables is. According to this scheme, variables are regarded as influential and very influential parameters in the equation if their P-values are less than 0.05 and 0.0001, respectively. C recovery is covered in Table 4, and IP is covered in Table 5. Table 4, Table 5 show that the temperature is extremely effective with a p-value of less than 0.0001.

**Table 4:** ANOVA analysis table and determination of effective parameters for C recovery.

Source	Sum of Squares	df	Mean Square	F-value	p-value
**Model**	5586.83	5	1117.37	46.23	<0.0001
A-Time (min)	109.18	1	109.18	4.52	0.0533
B-Temperature (C)	4267.98	1	4267.98	176.57	<0.0001
AB	25.36	1	25.36	1.05	0.3243
A^2^	1.04	1	1.04	0.0431	0.8388
B^2^	508.22	1	508.22	21.03	0.0005
**Residual**	314.23	13	24.17		
**Cor Total**	5901.06	18

**Table 5:** ANOVA analysis table and determination of effective parameters for IP recovery.

Source	Sum of Squares	df	Mean Square	F-value	p-value
**Model**	4426.79	5	885.36	26.32	<0.0001
A-Time (min)	182.64	1	182.64	5.43	0.0366
B-Temperature (C)	3476.57	1	3476.57	103.35	<0.0001
AB	45.80	1	45.80	1.36	0.2642
A^2^	7.87	1	7.87	0.2341	0.6366
B^2^	59.07	1	59.07	1.76	0.2079
**Residual**	437.30	13	33.64		
**Cor Total**	4864.09	18			

**Fig. 4:**
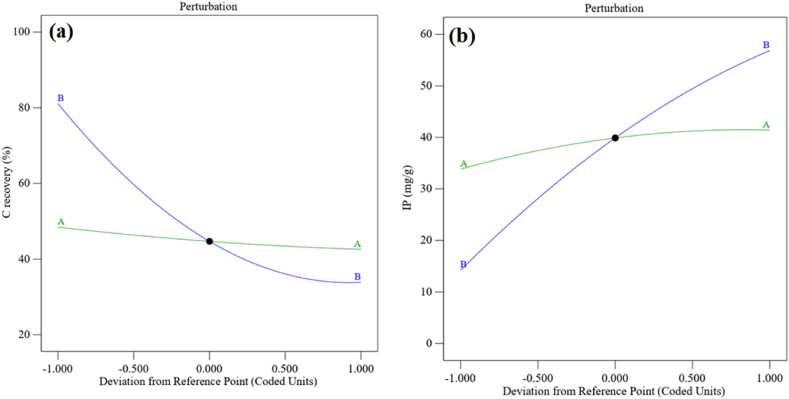
Effect of input parameters on response (a) C recovery and (b) IP recovery.

**Fig. 5:**
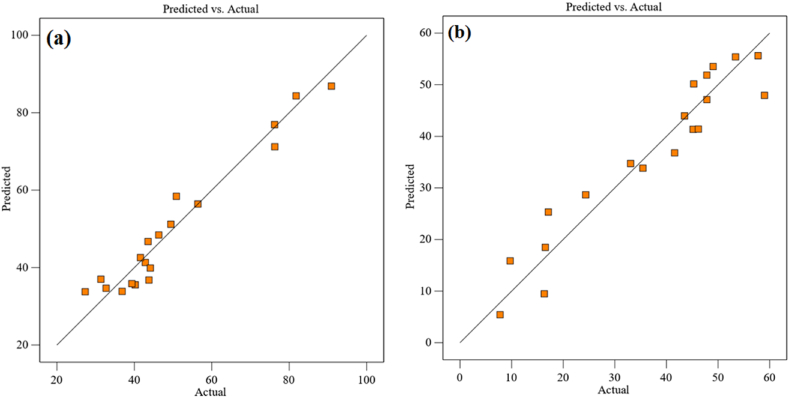
Actual data versus predicted data in RSM for (a) C recovery and (b) IP recovery.

The correlation with the amount of residual error and the model is measured using F-values. Model accuracy is shown by a high F-value and a modest p-value. As a result, the above equations illustrate the concept's importance. The interference diagram for the input factors influencing both replies' predictions is displayed in Fig. 4. Fig. 4 (a) reveals that the temperature parameter has a higher and negative trend that negatively affects the C recovery, whereas Fig. 4 (b) shows a steeper and positive slope that somewhat affects the IP recovery. The recovery rate is not significantly impacted by curves with a nearly constant, horizontal slope. The relationship amongst the real and anticipated values in Fig. 5(a and b) can be used to assess how well the model fits the data. Strong fits are indicated by models that have substantial R^2^ values. The statistical values derived by RSM for the two responses are shown in Table 6. A good match for the equation is shown by the large R^2^ value and the discrepancy within the Adjusted R^2^ and Predicted R^2^ values of below 0.1. Table 6 shows that the precision of the Adeq parameter is 19.2203 for C recovery and 15.4072 for IP, both of which are beneficial.

**Table 6:** Comparison of RSM statistical values for two responses.

Recovery	R^2^	Adj R^2^	Pred R^2^	Adeq Precision	Std. Dev	Mean	C.V. %
**C (%)**	0.9467	0.9263	0.8720	19.2203	4.92	50.10	9.81
**IP (mg/g)**	0.9101	0.8755	0.8121	15.4072	5.80	36.68	15.81

#### 3.1.1 Fitting the regression model for C and IP recovery in HTC

After analyzing the ANOVA table, another valuable piece of information is the predictive equation for the responses [49]. Obtainable from Eq. (12), Eqs. (13), (14)) show how different input variables affect responses received:

(13) $$C\;recovery\;\left(\%\right)=+241.88363-0.048454*\left(Time\right)-1.36823*\left(Temperature\right)+0.000121*\left(Time*Temperature\right)+0.000016*\left({Time}^{2}\right)+0.002272*\left({Temperature}^{2}\right)$$

(14) $$IP\;recovery\;\left(\frac{mg}{g}\right)=-79.794400.076111*\left(Time\right)+0.674215*\left(Temperature\right)-0.000162*\left(Time*Temperature\right)-0.000045*\left({Time}^{2}\right)-0.000775*\left({Temperature}^{2}\right)$$

Regression analysis in the RSM scheme utilizing data collected from experiments was used to find the model's variables based on the formulas that were supplied. As previously demonstrated, in this calculation, antagonistic effects are indicated by negative values, and synergistic effects are indicated by values that are positive.

### 3.2 Optimization of ANN

**Fig. 6:**
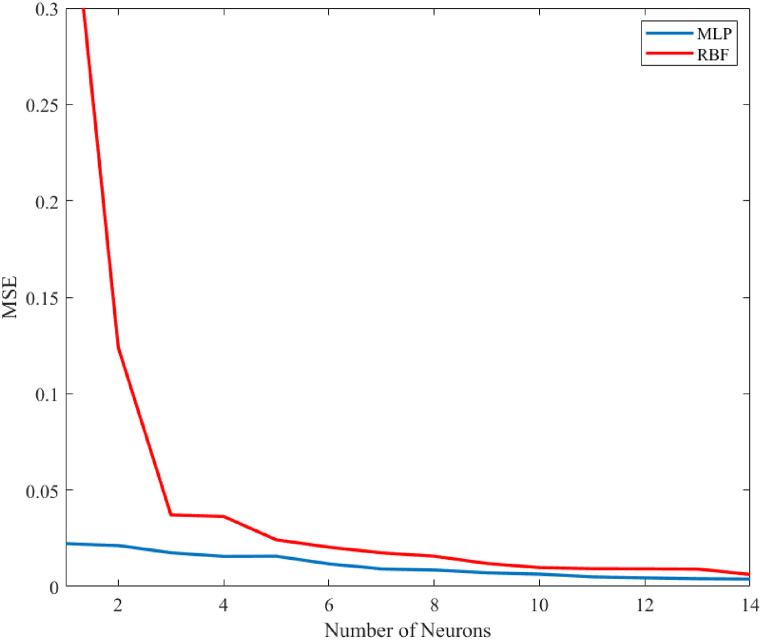
MSE graph based on the MLP and RBF's optimum neuron count.

**Fig. 7:**
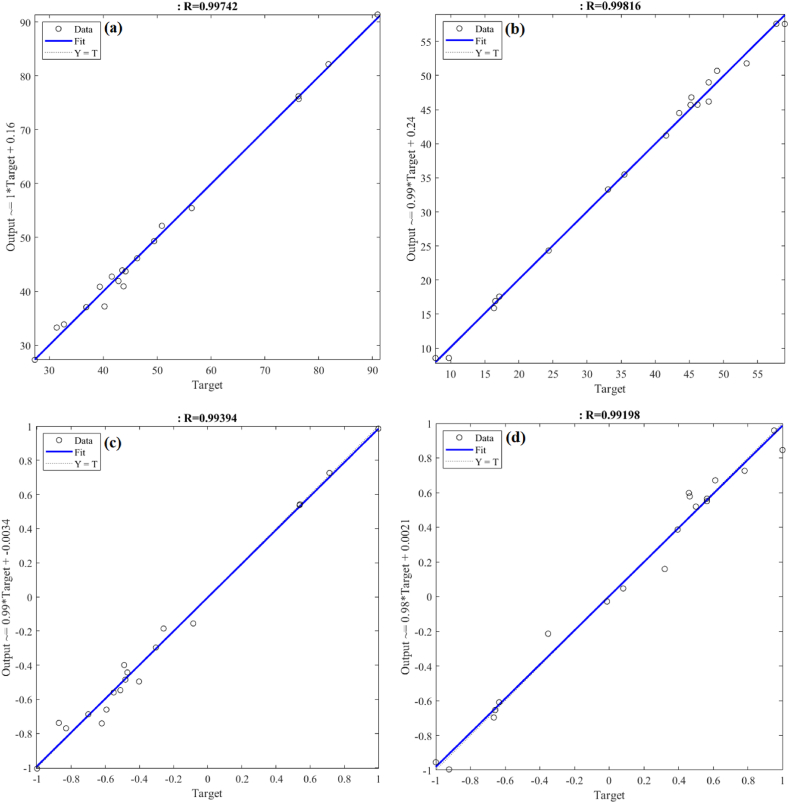
Comparison of MLP and RBF model predictions using datasets for (a) C recovery by MLP, (b) IP recovery by MLP, (c) C recovery by RBF and (d) IP recovery by RBF.

**Fig. 8:**
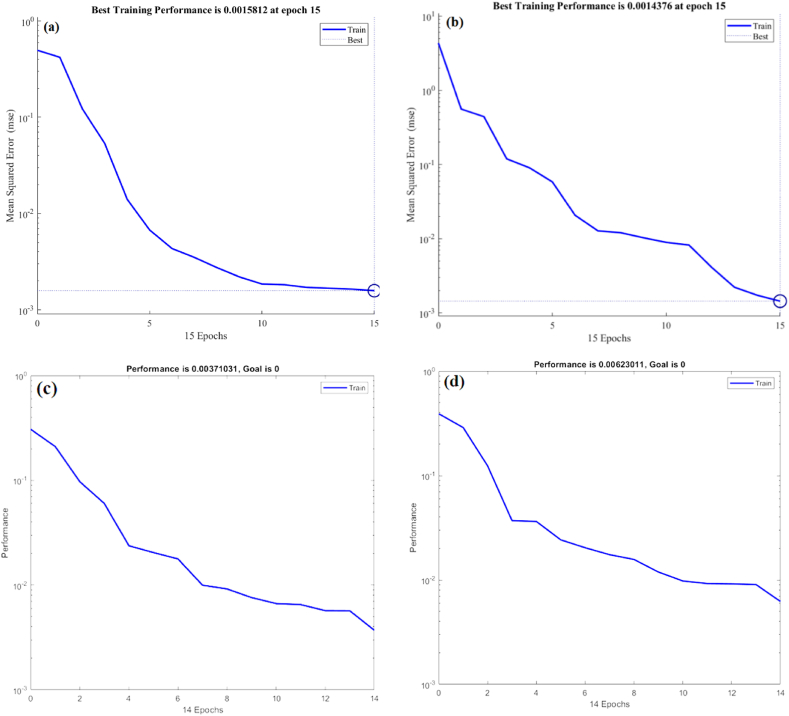
Performance comparison of MLP and RBF model predictions using datasets for (a) C recovery by MLP, (b) IP recovery by MLP, (c) C recovery by RBF and (d) IP recovery by RBF.

MLP and RBF neural networks were used in this investigation. MLP employed the Pureline transform function for the output layer and the Sigmoid transfer mechanism for the layers that were hidden. To find the proper MLP structure, the trial-and-error method has been employed [56]. The spectrum of neurons employed for MLP and RBF is 1–14, as shown in Fig. 6. The ideal quantity of neurons for an MLP is seven in two layers, as illustrated in Fig. 7, and for an RBF network, it is fourteen in one layer. Fig.8 illustrates a comparison of the performance results of MLP and RBF neural networks in terms of the optimal number of neurons. It is evident that MLP outperforms RBF, requiring fewer neurons for accurate predictions.

In order to get the best possible predicted accuracy with the least amount of model complexity, the number of neurons and layers in the ANN model was chosen using an iterative trial-and-error process. In order to reach the desired Mean Squared Error (MSE) for testing data, the work started with the fewest possible neurons and a single hidden layer. The neurons and layers were then progressively increased. Achieving a balance between accuracy and overfitting risk was one of the factors used to choose the final configuration. Another was making sure the model was sophisticated enough to identify the underlying patterns in the data without being overly complicated. This method improved the model's generalizability by enabling the development of an ideal architecture that avoided overfitting and lowered MSE.

#### 3.2.1 Prediction of C and IP recovery in HTC by ANN

To predict and equalize the experiment findings, a model for C and IP recovery in MLP is constructed using the Levenberg-Marquardt and RBF model methods [57]. Fig. 7(a–d) displays the outcomes of the ANN's MLP and RBF models. Experiment data and predicted models match exactly, as indicated by the strong R^2^ value. It is clear from comparing the MLP and RBF models that the MLP model can reproduce the experimental data more correctly even though it has less neurons. According to Fig. 7(a and b), the MLP model had extremely high R^2^ values, with R^2^__C recovery_ = 0.99742 and R^2^__IP recovery_ = 0.99816. Fig. 7 (c and d) show the RBF model's R^2^ values which were marginally lower, with R^2^__C recovery_ = 0.99394 and R^2^__IP recovery_ = 0.99198. This implies that the MLP model fits the experimental data more accurately than the RBF model.

Fig. 8(a–d) compare the predictions of the MLP and RBF models in terms of performance. With values of 0.0015812 for C recovery (Fig. 8(a)) and 0.0014376 for IP recovery (Fig. 8 (b)), the MLP model performed better than the others. However, with 0.00371031 for C recovery (Fig. 8 (c)) and 0.00623011 for IP recovery (Fig. 8 (d)), the RBF model performed better. These findings show that the MLP model performs more effectively than the RBF model, as evidenced by its lower performance values, in addition to offering a superior fit (higher R^2^ values).

### 3.3 Effects of parameter interactions

#### 3.3.1 Effect of temperature and time on C recovery

**Fig. 9:**
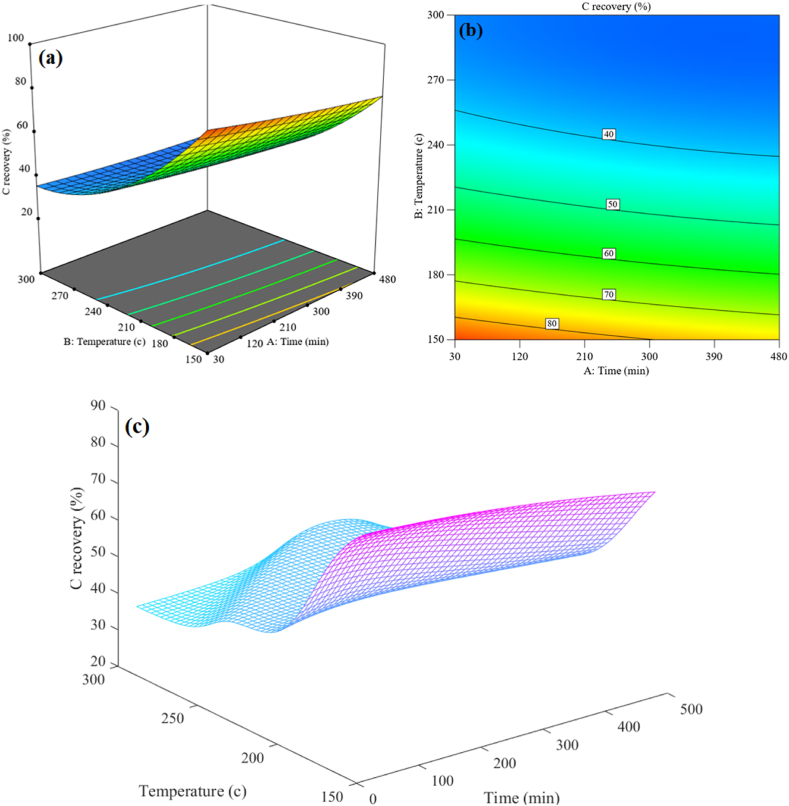
Comparison of the interaction effect of temperature and time on the C recovery (a) and (b) for RSM and (c) Predicted by MLP.

After examining the factors affecting the answer and presenting the model, it becomes important to consider how the answers change based on the inputs. In this section, 2D and 3D graphs were generated from RSM and subsequently compared with MLP neural network [63]. Fig. 9(a–c) shows the interaction effect of temperature and reaction time on C recovery. It can be seen that by reducing both temperature and time parameters, the amount of C recovery will increase. These results were compared with the results of MLP in Fig. 9 (c), which indicated that MLP is capable of making better predictions. Also, a similar outcome can be derived from the two-dimensional contours shown in Fig. 9 (b), where the yellow and orange regions indicate the highest response levels.

Light carbon-based molecules dissolving in a solution of water and then evaporate into organic gases are the causes of the drop in C content with increasing temperature and time. The processes of decarboxylation and dehydration also contribute to the reduction of the hydrocarbon's carbon content. The amount that is recovered of solid carbon decreases with increasing temperature and time of evaporation of carbon-based compounds. It has been discovered that carbon, which makes up the majority of the initial biological composition of PL, is decarboxylated throughout a procedure where the rate of C recovery falls off linearly with heating.

#### 3.3.2 Effect of temperature and time on IP recovery

**Fig. 10:**
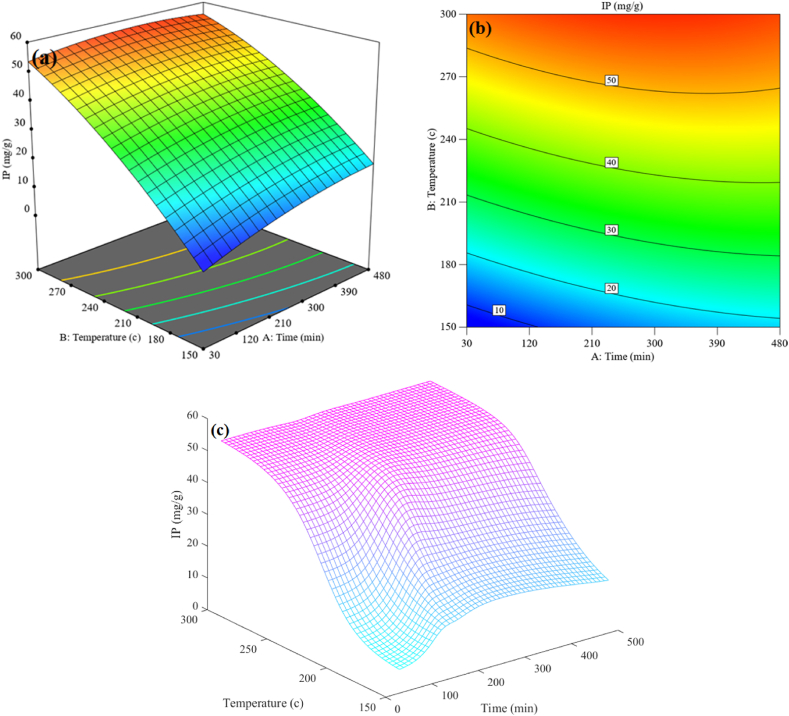
Comparison of the interaction effect of temperature and time on the IP recovery (a) and (b) for RSM and (c) Predicted by MLP.

Fig. 10 also presents another comparison between RSM and MLP for IP recovery. According to the three-dimensional Fig. 10 (a) and 10 (c), it can be observed that as temperature and reaction time increase, the IP recovery amount increases significantly. Also, the sharp increase in the two-dimensional contour presented in Fig. 10 (b) indicates that the orange and yellow areas represent the maximum amount of IP recovery. There is a decrease in tangible carbon left in the recycled coal as a result of the production of additional explosive hydrocarbons at greater temperatures. As a result, more inorganic phosphorus is recovered from PL. Because other components, like O and C, are reduced in the resultant hydrochar, IP retrieval increases with longer stay time and higher temperature. It is well known that, particularly for IP recovery, reaction time works better at lower temperatures than at higher ones. As shown in Fig. 10(c), the IP recovery rose by 65 % at a temperature of 150(c) from 9.5 (mg/g) in 30 (min) to 15.69 (mg/g) in 480 (min). A 13.7 % increase in IP recovery was seen at 300 (c), going from 51 mg/g in 30 min–58 mg/g in 480 min.

#### 3.3.3 Mechanisms underpinning how temperature and reaction time affect HTC's C and IP recovery

Numerous chemical and physical processes can be responsible for the reported effects of temperature and reaction time on the recovery of C and IP during HTC. The preservation of carbonaceous structures is probably the cause of the rise in carbon recovery with decreasing temperature and shorter reaction periods. Less carbonization and volatilization occurs at lower temperatures and over shorter periods of time, which results in less organic matter decomposition. This leads to a larger carbon recovery because more carbon stays in the solid phase. This event emphasizes how HTC processes need moderate conditions to maximize carbon retention [64]. On the other hand, IP recovery is improved by raising the temperature and reaction time. Phosphorus is released and converted into inorganic forms when organic phosphorus molecules are broken down by high temperatures and extended periods of time. These circumstances promote the mineralization and hydrolysis processes that raise IP's availability in the solid product. Higher temperatures may also encourage the transformation of phosphorus into inorganic forms that are thermodynamically stable, which would improve IP recovery even more [65]. All things considered, these findings demonstrate the different chemical routes that are triggered by different HTC circumstances. While higher temperatures and longer times encourage phosphorus mineralization, which leads to the effective recovery of, lower temperatures favor carbon preservation. This knowledge provides insightful information about how to best optimize HTC conditions for particular recovery goals [66].

### 3.4 Optimization of temperature and reaction time for C and IP recovery

The hyperparameters of ANN, such as the learning rate, momentum, number of neurons in hidden layers (NHL), and number of epochs, are optimized using Bayesian Optimization (BO). To reduce prediction error, as indicated by the Root Mean Square Error (RMSE), these hyperparameters are adjusted iteratively. The number of neurons in hidden layers varies from 10 to 100, the learning rates are adjusted between 0.01 and 1, and The desired response is attained in 15 epochs, with an epoch count ranging from 10 to 1,000 [[67], [68], [69]]. Optimizing the experimental data to achieve the best response under the optimal input operating conditions is one of the most valuable studies to be addressed. Optimization was performed using RSM software within the specified operating range of temperature (150–300 (c)) and time (30–480 (min)) for C recovery and IP. According to Table 5, Table 7 repetitioTable 5, Table 7ns have been conducted, and the software suggested one of the best modes. By examining these values, it can be observed that under the operating conditions of a temperature of 182.329 (c) and a time of 427.746 (min), the C recovery and IP values were determined to be 59.611 % and 29.114 (mg/g), respectively.

**Table 7:** Optimum values of input parameters and amount of C and IP recovery.

Num	Time (min)	Temperature (c)	IP recovery (mg/g)	C recovery (%)	Desirability	
**1**	**427.746**	**182.329**	**29.114**	**59.611**	**0.460**	**Selected**
2	429.419	182.215	29.091	59.646	0.460	
3	30.000	300.000	53.534	35.560	0.341	
4	30.003	298.477	53.221	35.567	0.340	
5	480.000	300.000	55.637	33.776	0.308	

### 3.5 Comparison of modeling strategies for IP and C recovery estimation

According to Table 8 three modeling techniques were used in this study to forecast the recovery of C and IP: RBF, MLP, and RSM. A number of factors, including accuracy, model complexity, adaptability, strengths and limitations, and validation methods such MSE values, were taken into consideration when evaluating each strategy. Both R^2^ values and MSE were used to evaluate each model's accuracy. With R^2^ values of 0.99742 for C and 0.99816 for IP, as well as matching MSE values of 0.0015812 for C and 0.0014376 for IP, the MLP model showed excellent predictive ability. These metrics demonstrate the MLP model's high degree of precision and low prediction error in capturing the underlying relationships in the dataset. The RBF network, on the other hand, yielded somewhat poorer accuracy, with R^2^ values that were similar to the MLP's but higher MSE values (0.0037103 for C and 0.00623011 for IP). The MLP's superior accuracy and dependability are further supported by the higher MSE values, which indicate a higher prediction error in the RBF model when compared to the MLP. R^2^ values from the RSM model were 0.9101 for IP and 0.9467 for C. Reduced prediction accuracy may arise from RSM's inability to adequately capture intricate, nonlinear relationships in the data, as seen by the relatively lower R^2^ values.

**Table 8:** Comparison between the values of R^2^ and the number of neurons in the three methods MLP, RBF and RSM.

Recovery	R^2^			Neurons		
	MLP	RBF	RSM	MLP	RBF	RSM
**C (%)**	0.99742	0.99394	0.9467	7	14	–
**IP (mg/g)**	0.99816	0.99198	0.9101	7	14	–

Regarding model complexity, the RBF model needed fourteen neurons to get similar predictive accuracy, whereas the MLP model only needed seven neurons to achieve excellent predictive accuracy. This difference demonstrates the MLP's effectiveness because it used fewer neurons to create lower MSE values, which decreased computational costs and reduced the possibility of overfitting. The RSM is computationally less expensive than neural networks because it is a polynomial-based model and does not use neurons. However, as seen by its lower R^2^ values, its simplicity limits its capacity to precisely forecast intricate, nonlinear interactions in HTC processes. The nonlinear patterns in the data were handled with flexibility by both the MLP and RBF models. The MLP model demonstrated good generalization for this application by adapting to the complicated data structure more effectively while using fewer neurons and achieving a lower MSE. The RBF model, on the other hand, is less flexible in modeling complex interactions, which reduces its predictive power for C and IP recovery, even though it works well in simpler applications.

Each model's performance measures show both its advantages and disadvantages. The MLP model is appropriate for complicated, nonlinear data because of its strengths, which include excellent accuracy and low MSE values attained with fewer neurons. Its vulnerability to overfitting, which can be lessened by regularization and validation strategies, is a possible drawback, though. High accuracy and adaptability are also displayed by the RBF model; nevertheless, it is less effective than the MLP due to its higher MSE values and larger neuron count, which suggest higher processing demands and greater prediction error. Although the RSM model is straightforward to understand and computationally efficient, its lower R^2^ values show that it has less predictive potential for nonlinear data because of its limited ability to capture complicated relationships. All models underwent cross-validation to guarantee their generalizability and robustness. Given its superior performance across both R^2^ and MSE metrics, these validation procedures verified that the MLP model is the most efficient method for precisely forecasting C and IP recovery. This updated research highlights the MLP model's effectiveness and predicted precision, which are bolstered by its low MSE values. While noting the shortcomings of the RBF and RSM models, the discussion offers a fair comparison of each model, emphasizing the MLP's advantages.

### 3.6 Evaluation of performance compared to other methods

Apart from comparing the MLP, RBF, and RSM models, it is crucial to evaluate how well these methods perform in comparison to other popular modeling approaches in biomass conversion and HTC processes. A popular method for regression tasks, support vector regression (SVR) has been employed in a number of domains, including biomass conversion. Although SVR is capable of capturing nonlinear relationships, the choice of kernel functions and hyperparameters has a significant impact on how well it performs. In contrast, the MLP model employed in this investigation showed better predictive accuracy, forecasting the recovery of C and IP with a reduced MSE. Using ensemble learning, Random Forest (RF) is a potent technique that produces excellent prediction performance. However, when used on huge datasets, it might encounter issues with interpretability and computing performance. According to the study's findings, the MLP model improved computing efficiency by using fewer neurons and outperforming Random Forest in terms of accuracy [70]. Despite the fact that ANN approaches are widely used in many different applications, their performance can be greatly impacted by the particular architecture and optimization techniques used. Compared to typical ANN architectures, which frequently require more extensive tuning and may not generalize well across different datasets, the MLP model used in this study showed excellent accuracy and efficiency. With better prediction accuracy and lower MSE values for both C and IP recovery, the MLP model outperformed the other methods overall. The MLP is a popular option for HTC process optimization due to its computing efficiency and ability to accurately predict intricate, nonlinear relationships. With its potential for broader use in modeling biomass conversion processes, this performance comparison highlights the importance of using MLP models to forecast recovery outcomes in HTC [71].

## 4 Conclusion

This study shows how well the ANN-based algorithm model predicts the recovery of C and IP in HTC processes when compared to RSM. Understanding the impacts of temperature and reaction time offers valuable insights that can be used to optimize HTC settings and achieve desired healing outcomes. To build on these discoveries and enable HTC's practical application on an industrial scale, more study is required. Through a comparison of the two models' 2D and 3D networks, it was found that the MLP outperforms the RSM and has a greater accuracy rate for point predictions. The modeling results clearly showed that temperature has a greater effect than time on the rate of both responses. Also, with the decrease in temperature and reaction time, the C recovery increased. Simultaneously, the recovery of IP increased significantly with higher temperatures and longer reaction times. Therefore, the profiles obtained from RSM and MLP showed a consistent increase in IP recovery and a consistent decrease in C recovery at elevated temperatures and durations. The reason for this is several mechanisms that occur simultaneously in the HTC reactor at various temperatures and durations. When the R^2^ coefficients for MLP and RSM were compared, it was found that MLP is more accurate than RSM, with an R^2^ value above 0.99. Although the value of R^2^ for both MLP and RBF models was close, it was observed that MLP with the optimal number of 7 neurons in two layers outperformed RBF with 14 neurons. Finally, within the specified data range, optimization operations were conducted on the C and IP recovery. It has been observed that at the optimal values of temperature and reaction time, which are 182.329 (c) and 427.746 (min) respectively, C recovery and IP values of 59.611 % and 29.114 (mg/g) were achieved.

### 4.1 Future research directions

• To obtain a more thorough grasp of their impacts on C and IP recovery, future research should look into other process variables such feedstock composition, pH levels, and catalyst addition. Examining these variables may result in improved recovery effectiveness and more sophisticated optimization techniques.
• Even though the current research provides insightful information at the laboratory scale, there are particular difficulties in scaling up HTC for commercial applications, such as material handling, energy needs, and reaction control. Future studies should concentrate on resolving these issues by creating affordable solutions and scalable designs for extensive HTC operations.
• There is a lot of promise for industrial uses for the recovered C and IP. IP recovery offers a sustainable supply of phosphorus for agricultural use, while carbon-rich hydrochars can be used as adsorbents, energy sources, or soil additives. To assess these goods' viability in practice and environmental advantages, research on their use, stability, and effects on the environment is crucial.
• Larger-scale implementation of an efficient HTC process will need resolving issues with economic viability, regulatory compliance, and interaction with current systems. To help HTC become a practical waste-to-resource technology, more study should evaluate these aspects and offer industry standards.

By facilitating the transfer of HTC from laboratory-scale research to real-world industrial applications, these future research avenues will increase the possibility of sustainable resource recovery and support circular economy initiatives.

## CRediT authorship contribution statement

**Abolfazl Shokri:** Writing – review & editing, Writing – original draft, Visualization, Validation, Investigation, Data curation, Conceptualization. **Mohammad Amin Larki:** Writing – review & editing, Formal analysis, Data curation, Conceptualization. **Ahad Ghaemi:** Writing – review & editing, Writing – original draft, Visualization, Validation, Supervision, Project administration, Methodology, Investigation, Funding acquisition, Formal analysis, Data curation, Conceptualization.

## Data availability statement

Data included in article.

## Declaration of competing interest

The authors declare that they have no known competing financial interests or personal relationships that could have appeared to influence the work reported in this paper.

**Table:** 

***Nomenclature Abbreviations***	
t	Time
T	Temperature (C)
C	Carbon
P	Phosphorous
R^2^	Correlation coefficient
ANN	Artificial Neural Network
RBF	Radial Basis Functions
MLP	Multilayer Perceptron
MSE	Mean Square Error
HTC	Hydrothermal Carbonization
HC	Hydrochar
IP	Inorganic Phosphorous
PL	Poultry litter
MSE	Mean Squared Error
***Subscript***	
x	Equation input
y	Equation output
w	Weight
b	Bias
σ	Basis width
i	Experiment set i
j	Experiment set j
k	Experiment set k
